# Assessment of Pain Types in Recently Diagnosed Patients With Inflammatory Arthritis

**DOI:** 10.1002/acr.25651

**Published:** 2025-10-07

**Authors:** Zoe Rutter‐Locher, Sam Norton, Joseph L. Taylor, Bina Menon, Tom Esterine, Ruth Williams, Leonie S. Taams, Kirsty Bannister, Bruce W. Kirkham

**Affiliations:** ^1^ Guy's and St Thomas’ NHS Trust London United Kingdom; ^2^ King's College University London United Kingdom; ^3^ Imperial College London London United Kingdom

## Abstract

**Objective:**

Up to 40% of patients with inflammatory arthritis (IA) experience persistent pain, traditionally thought to be associated with a shift from peripherally to centrally mediated pain during the disease course in some patients. We assessed sensory profiles of recently diagnosed individuals with IA, hypothesizing that pain reported at this early stage of diagnosis is driven predominantly by peripheral joint inflammation.

**Methods:**

Recently diagnosed patients with IA with pain numerical rating scale scores of ≥3 were recruited. We collected data on the following: arthritis activity (Disease Activity Score in 28 joints [DAS28], musculoskeletal ultrasonography), quality of life (Musculoskeletal Health Questionnaire [MSK‐HQ], EuroQol 5‐domain), mental health status (Patient Health Questionnaire Anxiety–Depression Scale [PHQ‐ADS]), and pain characteristics (fibromyalgia criteria, painDETECT, static and dynamic quantitative sensory testing [QST]).

**Results:**

Sixty‐one participants (57% female, 62% with rheumatoid arthritis) were enrolled (mean ± SD age 49.8 ± 15 years; mean ± SD time since diagnosis 1.2 ± 2.3 months). Ninety‐seven percent had peripheral joint inflammation, with a mean ± SD DAS28 score of 3.8 ± 1. However, 21% met the fibromyalgia criteria, 25% had a painDETECT score of ≥19, and 20% had a tender joint count minus swollen joint count of ≥7, which significantly correlated with DAS28, MSK‐HQ, and PHQ‐ADS scores. QST revealed lowered pressure pain thresholds at nonarticular sites in a subset of participants and facilitated temporal pain summation and deficient pain modulation in 18% and 61% of patients, respectively.

**Conclusion:**

This study provides evidence of centrally mediated pain at the time of diagnosis, challenging the notion that, even at the early stage of disease, pain is driven only by peripheral mechanisms.

## INTRODUCTION

Despite major improvements in therapies and treatment strategies, up to 40% of patients diagnosed with inflammatory arthritis (IA), including rheumatoid arthritis (RA), psoriatic arthritis, and ankylosing spondylitis, continue to experience persistent pain.^1^, ^2^, ^3^, ^4^ Pain intensity initially improves during the six‐month period post diagnosis, likely attributable to the anti‐inflammatory properties of disease‐modifying antirheumatic drugs. However, after that period, joint tenderness and pain persist, which might be unresolved inflammation, or as often found in the absence of synovitis, it might be pain sensitization^1^ or a combination of both.^5^ A recent meta‐analysis by our group found that, according to patient‐reported outcome measures (PROMs), pain sensitization was present in 20% to 30% of individuals with established IA, with the largest group being those with psoriatic arthritis.^6^ What underlies this pain sensitization remains equivocal, with postulated mechanisms encompassing dysfunction in both peripheral and central pain‐processing pathways, including spinal and supraspinal circuits.^7^, ^8^, ^9^, ^10^ Improving our understanding of how persistent pain is initiated, develops, and is maintained in the patient population with IA is crucial because it could help guide appropriate therapeutic strategies.

**SIGNIFICANCE & INNOVATIONS**

- This study is the first to record comprehensive sensory profiles incorporating clinical examination, ultrasonography, questionnaire, and quantitative sensory testing (QST) data in the early stage of inflammatory arthritis diagnosis.
- Of those tested, 97% had peripheral joint inflammation confirmed by clinical assessment and ultrasonography. In addition, clinical, patient‐reported, and QST outcome measures revealed the likely presence of centrally mediated pain in a subset of participants.
- These findings challenge the longstanding assumption that only peripheral mechanisms driven by joint inflammation underpin pain at diagnosis, highlighting a role for centrally mediated pain even in the early stage of the disease.

Pain in IA can have many different causes, ranging from persistent joint inflammation to dysfunctional neural processes, which require the use of multiple assessment tools to provide a more complete understanding. Musculoskeletal ultrasonography (MSKUS) provides an objective assessment of peripheral joint inflammation in addition to clinical examination, whereas quantitative sensory testing (QST) allows activity in peripheral nerves to be inferred (static QST) alongside the functionality of central pain‐processing pathways (dynamic QST). When combined with validated questionnaire data, which provide insights into the patient pain experience, clinical examination, MSKUS, and QST outcomes can be used to build an individual's sensory profile. Cumulatively, these profiles can help deduce likely pain‐causing mechanisms.^11^, ^12^

Studies using questionnaire and QST methods, mainly cross‐sectional design in patients with longstanding IA,^13^, ^14^ have suggested the presence of centrally mediated pain in a subset of patients. This research has led to the current consensus that, in some patients, during their disease course, pain primarily caused by peripheral joint inflammation at diagnosis transforms to include pain that is underpinned by dysfunction in central nervous system processes.^7^ The temporal nature of this shift during the disease course remains unclear because no studies have explored detailed pain characteristics at diagnosis, and there are limited longitudinal studies. In keeping with the current understanding that centrally mediated pain develops over the course of the disease, we hypothesized that peripherally mediated inflammatory pain would predominate during this initial disease phase, with little evidence of dysfunctional central pain processing. Here we report the initial sensory profiles of individuals with IA promptly after diagnosis, established with an array of techniques encompassing both MSKUS and QST alongside validated questionnaires. To our knowledge, this is the first study to employ such a detailed multimodal approach to sensory profiling in newly diagnosed patients with IA.

## PATIENTS AND METHODS

### Study population

This study presents baseline data obtained from an initial cohort of 61 patients enrolled in the Pain Phenotypes and Their Underlying Mechanisms in Inflammatory Arthritis Study (PUMIA). PUMIA is a single‐site, prospective, longitudinal, observational study aiming to understand pain mechanisms in IA. Recruitment of participants took place at the rheumatology outpatient department of Guy's Hospital, London, between February 2022 and November 2022. Inclusion criteria for the study encompassed individuals who reported a total numerical rating scale (NRS) pain score of 3 or higher on a scale of 0 to 10, received a clinician diagnosis of peripheral inflammatory joint disease, and experienced symptom onset within the past 12 months. Patients were enrolled in the study as soon as feasible following their diagnosis.

Patients were excluded if they were under 18 years of age, were unable or unwilling to provide informed written consent, were unable to adhere to study protocols, were pregnant or breastfeeding, were receiving non‐IA–related immunosuppressant therapy, were undergoing current or recent (within the last 90 days) treatment with investigational agents, or had a history of severe peripheral vascular disease or peripheral neuropathy. Use of opioids, gabapentin, or pregabalin was stopped 24 hours before assessment. All participants gave fully informed written consent, and the study protocol received approval from Bromley Research Ethics Committee and the Health Research Authority (research ethics committee number 21/LO/0712).

### Clinical assessment and ultrasonography

Routine demographic data (including age, sex, ethnicity—self‐reported from a predefined list—body mass index, smoking status, employment status, and comorbidities), disease data (including diagnosis, disease duration, and serology), and medication history were obtained from each participant. The term “current steroids” refers to oral prednisolone taken within the past two weeks or intramuscular methylprednisolone acetate administered within the last three months.

Patients were assessed for disease activity (C‐reactive protein [CRP], 68 tender joint count [TJC], 66 swollen joint count [SJC], Disease Activity Score in 28 joints [DAS28], TJC − SJC using 28 joints) and completed questionnaires for quality of life (QoL; Musculoskeletal Health Questionnaire, EuroQol 5‐domain^15^), impact of disease (Rheumatoid Arthritis Impact of Disease^16^), somatic symptoms (Patient Health Questionnaire 15 [PHQ‐15]^17^), and mental health (PHQ‐9 for depression,^18^ Generalized Anxiety Disorder 7 [GAD‐7]^19^). Fibromyalgia status was determined according to the 2016 revision of the American College of Rheumatology 2010 modified fibromyalgia diagnostic criteria^20^ (Widespread Pain Index [WPI] and Symptom Severity Scale [SSS] score). Fibromyalgia severity was assessed as the sum of the WPI and SSS scores. Pain assessment was conducted using (1) the NRS for, pain in which individuals are asked to score, between 0 and 10, both total body pain and most painful joint pain in the last 24 hours (the “target joint”), and (2) the painDETECT questionnaire, which identifies neuropathic‐like pain.^21^ The painDETECT has been used in previous studies as a proxy for pain caused by central mechanisms due to the similarity in symptom profiles.^22^, ^23^, ^24^

A 14‐joint MSKUS assessment was used to obtain an objective evaluation of joint inflammation, particularly subclinical synovitis. Bilateral radiocarpal joints, intercarpal joints, ulnocarpal joints, metacarpophalangeal joints 2 and 3, proximal interphalangeal joints 2 and 3, and metatarsophalangeal joints 2 and 3 were graded semiquantitatively for gray scale (GS) and power Doppler (PD) according to the EULAR–Outcome Measures in Rheumatology criteria^25^ by two individual raters, blinded to patient demographics or sensory profile.

### QST

#### Static QST

The QST protocols established by the German Research Network on Neuropathic Pain (DFNS) were applied to assess cutaneous sensory detection using standardized von Frey hairs (mechanical detection threshold [MDT]) and pain thresholds using a standardized pinprick set (mechanical pain threshold [MPT]) on the target most painful joint and control site (contralateral volar forearm).^26^ These tests are performed using standardized instructions in which normative data, grouped by age, sex, and site, are available to allow comparisons with healthy controls.^27^ The established DFNS protocol was also used to assess the pressure pain threshold (PPT) at the joint line of the target most painful joint, the dorsal aspect of bilateral wrists, and a nonarticular control site (bilateral trapezius muscles) using a Wagner Force 25 FDX algometer (Wagner Instruments).^26^ PPT is the point at which a pressure applied first becomes painful, and lower pain thresholds reflect higher pain sensitivity. Lower PPT at nonarticular sites suggest widespread pain likely underpinned by central mechanisms, whereas lower PPT at joint sites only suggests local joint pain sensitivity due to joint inflammation and peripherally mediated mechanisms.

#### Dynamic QST

Dynamic QST comprises temporal summation of pain (TSP) and conditioned pain modulation (CPM) paradigms. The TSP paradigm provides a proxy measure of spinal neuronal activity. In brief, delivery of an equal‐intensity noxious stimulus above a critical rate leads to an increase in the individual's perception of that stimulus. We used two (TSP) paradigms. The first followed a DFNS protocol using pinprick stimulators,^26^ and the second followed a standard protocol using cuff pressure algometry (detailed protocol in the Supplementary Material). Spinal neuronal activity can be modulated through descending modulatory circuits, whose function can be assessed upon application of a CPM paradigm.^28^, ^29^ In brief, a measure of painfulness for a test stimulus is taken, in the absence or presence of a second painful “conditioning” stimulus. The difference in the participant's ratings of the test stimuli (before and during conditioning) is the measure of a “CPM effect,” in which a “CPM responder” refers to an individual who experiences a significant reduction in pain rating of the test stimulus upon conditioning whereas a “CPM nonresponder” does not. For the CPM paradigm, we followed a computerized cuff algometry protocol.^30^

### Statistical analysis

All data are presented as mean ± SD. Paired or independent *t*‐tests were employed, as appropriate, to identify significant differences in means. To normalize the data for MDT, MPT, PPT, and TSP, logarithmic transformations were applied,^27^, ^31^ resulting in values with a mean of 0 and an SD of 1. Absolute reference data (matched for age, sex, and site—dorsum of the hand for MDT, MPT, and TSP and thenar eminence for PPT) were used to normalize test results of the individual participant by calculating the Z transformation: (value participant − mean controls)/SD controls.^32^ A Z score of ±1.96 corresponded to statistical significance at the 0.05% level.

In addition to descriptive analysis, to provide a more formal test of the relative contribution of inflammation and other candidate variables to pain, we conducted a regression model with pain NRS as the dependent variable. MSKUS total GS and PD; our preferred measure of peripheral inflammation) was entered in the first block, followed by demographic factors in the second block to estimate the adjusted association, followed by other plausible contributors to pain, including fatigue, mood, and centrally mediated pain markers, in a third block. This blockwise approach allowed us to estimate the variance in pain explained by inflammation alone and the incremental contribution of other factors.

Pearson correlations were used to investigate the relationships between markers of central pain mechanisms, peripheral inflammation, and QST parameters and clinical outcomes, such as pain levels, disease activity, and mental health. All assumptions for parametric tests were checked before analysis. For variables showing deviation from normality, key analyses were repeated using nonparametric equivalents (eg, Spearman's correlation) as a sensitivity check. Given the exploratory nature of this analysis, effect sizes were primarily evaluated. For all analyses, *P* values below 0.05 were considered statistically significant.

Finally, an exploratory factor analysis was conducted to identify underlying latent variables (factors) accounting for the common variance across observed variables (detailed protocol in the Supplementary Material). All analyses were performed using Stata version 17.0 (StataCorp LLC). All data are available upon request to the corresponding author.

## RESULTS

### Demographics and clinical assessment

#### Demographics

We recruited 61 patients composed of a broad range of ethnicities predominantly with a diagnosis of RA (62%), with the remaining 38% having a mixture of early IA, psoriatic arthritis, and spondylarthritis. The mean age of participants was 49.8 years (range 19–86 years), and most participants were female (57%). Mean ± SD symptom duration was 6.3 ± 3.1 months, and mean ± SD time since diagnosis was 1.2 ± 2.3 months (Table 1).

**Table 1:** Demographic and pain data*

Variable	Value
Demographic (N = 61)	
Age, mean ± SD (range), years	49.8 ± 15.4 (19–86)
Female sex, n (%)	35 (57)
Ethnicity, n (%)	
White British	33 (54)
Black or Black British	11 (18)
Asian or Asian British	7 (12)
Other	10 (16)
Smoker, current or previous, n (%)	23 (38)
Unemployed, n (%)	9 (15)
BMI, mean (SD)	26.8 (5.7)
Diagnosis	
EIA, n (%)	16 (26)
RA, n (%)	38 (62)
PsA, n (%)	6 (10)
SpA, n (%)	1 (2)
Seropositive: RF or CCP, n (%)	37 (61)
Time since diagnosis, mean (SD), months	1.2 (2.3)
Symptom duration, mean (SD), months	6.3 (3.1)
DAS28‐CRP, mean (SD)	3.8 (1.0)
TJC‐28, mean (SD)	6.5 (5.2)
SJC‐28, mean (SD)	2.7 (2.5)
Steroid and DMARDs, n (%)	
Steroids in some form at assessment	37 (61)
IM depomedrone in last 3 months	25 (41)
PO prednisolone in last 2 weeks	13 (21)
IV methylprednisolone	2 (3)
cDMARDs	36 (59)
Monotherapy	33 (54)
Combination therapy	3 (5)
Methotrexate	32 (52)
Quality of life, mean (SD)	
MSK‐HQ	29 (11)
EQ‐5D	0.58 (0.28)
EQ‐5D total health VAS	59 (22)
RAID	4.7 (2.6)
Mental health	
GAD‐7, mean (SD)	7.4 (6.0)
Anxiety total, score ≥5, n (%)	38 (62)
Mild anxiety, score 5–9, n (%)	21 (34)
Moderate anxiety, score 10–14, n (%)	7 (11)
Severe anxiety, score ≥15, n (%)	10 (16)
PHQ‐9, mean (SD)	8.1 (6.4)
Depression total, score ≥5, n (%)	40 (66)
Mild depression, score 5–9, n (%)	17 (28)
Moderate depression, score 10–14, n (%)	13 (21)
Moderately severe depression, score 15–19, n (%)	7 (11)
Severe depression, score ≥20, n (%)	3 (5)
PHQ‐15, mean (SD)	9.0 (5.5)
Somatic symptoms total, score ≥5, n (%)	49 (80)
Mild somatic symptoms, score 5–9, n (%)	25 (40)
Moderate somatic symptoms, score 10–14, n (%)	15 (24)
Severe somatic symptoms, score ≥15, n (%)	9 (15)
Pain, mean (SD)	
Total body pain	5.5 (2.1)
Target joint pain	5.1 (2.5)
Widespread pain	
WPI score, mean (SD)	5.6 (2.9)
WPI, score of ≥7, n (%)	19 (31)
Fibromyalgia, n (%)	13 (21)
TJC − SJC, score of >7, n (%)	12 (20)
painDETECT	
painDETECT, mean (SD)	12.7 (8.0)
Likely neuropathic‐like pain, score of ≥19, n (%)	15 (25)
Possible neuropathic‐like pain, score between 13 and 18, n (%)	9 (15)
Unlikely neuropathic‐like pain, score ≤12, n (%)	37 (61)

* Table showing patient demographics, diagnosis, and medication alongside results of questionnaire data collected for quality of life, mental health, fibromyalgia criteria, and painDETECT (N = 61). BMI, body mass index; CCP, cyclic citrullinated peptide; cDMARD, conventional disease‐modifying antirheumatic drug; DAS28‐CRP, Disease Activity Score in 28 joints using the C‐reactive protein level; DMARD, disease‐modifying antirheumatic drug; EIA, early inflammatory arthritis; EQ‐5D, EuroQol 5‐domain; GAD‐7, Generalized Anxiety Disorder 7; IM, intramuscular; IV, intravenous; MSK‐HQ, Musculoskeletal Health Questionnaire; PHQ, Patient Health Questionnaire; PO, oral; PsA, psoriatic arthritis; RA, rheumatoid arthritis; RAID, Rheumatoid Arthritis Impact of Disease; RF, rheumatoid factor; SJC‐28, swollen joint count for 28 joints; SpA, spondylarthritis; TJC‐28, tender joint count for 28 joints; TJC − SJC, tender joint count minus swollen joint count; VAS, visual analog scale; WPI, Widespread Pain Index.

#### Disease activity and therapy

The mean ± SD DAS28‐CRP was 3.8 ± 1. The mean ± SD total and target joint pain on an NRS (0–10) was 5.5 ± 2.1 and 5.1 ± 2.5, respectively (Table 1).

#### Mental health and QoL

**Figure 1:**
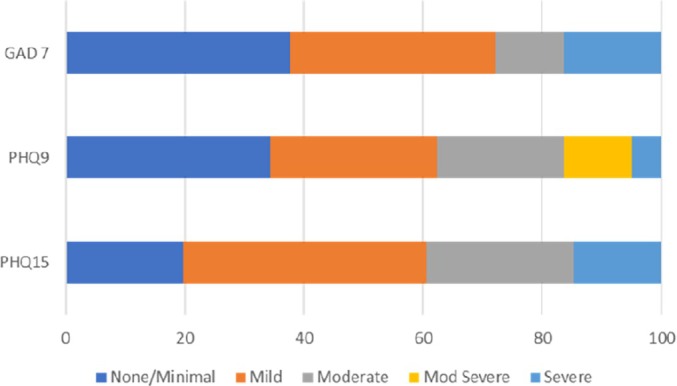
Prevalence of anxiety (GAD 7), depression (PHQ9), and somatic symptoms (PHQ15) in the study population (N = 61). GAD 7, Generalized Anxiety Disorder 7; PHQ, Patient Health Questionnaire.

The prevalence of participants with patient‐reported outcome indications of anxiety, depression, and somatization in the patient population was high (Table 1 and Figure 1). QoL was in keeping with established RA populations.^33^

#### Ultrasonographic assessment

There was evidence of active synovitis either clinically or on ultrasonography in 59 patients (97%), in keeping with a recent IA diagnosis. Of the 59, 37 (61%) had GS positivity, and 22 (36%) had PD positivity at the target joint (Table 2). Of the 24 patients (39%) who had neither GS or PD positivity at the target joint, 17 had GS or PD positivity at other joints, and 7 did not, although 5 of those had clinically swollen joints not examined by ultrasonography. Of note, 24 of the 39 patients with PD 0 at the target joint had recently received steroid therapy, which may have suppressed the PD signal.

**Table 2:** GS and PD activity on musculoskeletal ultrasonography of the target most painful joint*

GS score	n (%)	PD score	n (%)
GS 0	24 (39)	PD 0	39 (64)
GS 1	16 (26)	PD 1	5 (8)
GS 2	18 (30)	PD 2	11 (18)
GS 3	3 (5)	PD 3	6 (10)

* n (%) values of patients with each score at the target joint. GS, gray scale; PD, power Doppler.

#### Clinical outcome measures and PROMs

The assessment of WPI and TJC − SJC provides an indication of widespread pain,^34^ which is often associated with centrally mediated pain. Of the total 61 participants, 20% to 30% fulfilled criteria for widespread pain, fibromyalgia, and neuropathic‐like pain on the painDETECT questionnaire (Table 1), and these PROMs were correlated (Supplementary Table S1).

### QST

In this section, we provide a summary of the QST findings. Further details for interested readers are presented in the Supplementary Material.

#### Static QST: cutaneous sensation and pain thresholds

**Figure 2:**
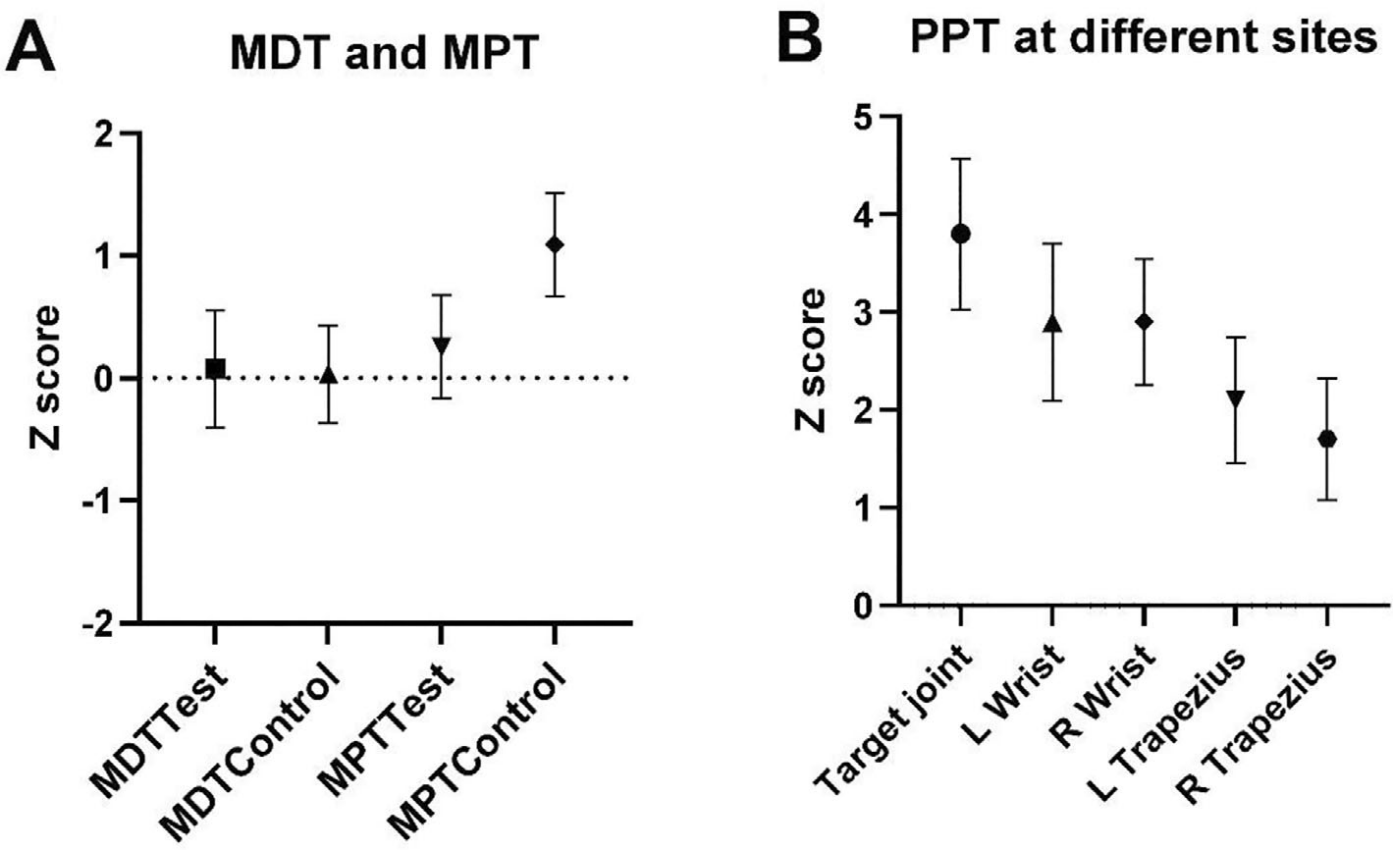
Graphs showing results from static QST. (A) Z score for the study population MDT and MPT at test (target joint) and control sites (contralateral forearm) (n = 61). The QST Z score chart illustrates the trend of sensory change in which positive Z scores suggest increased sensory capability (ie, hypersensitivity), whereas negative scores point to diminished sensory ability (ie, hyposensitivity). There is no significant difference in the MDT or MPT compared to normative data, reflected by Z scores <1.96. (B) Z score for PPT at each site (n = 60). As a group, participants showed significantly increased sensitivity at all sites, except the right trapezius (mean PPT at target joint 3.8 [SD 3.0], left wrist 2.9 [SD 3.1], right wrist 2.9 [SD 2.5], left trapezius 2.1 [SD 2.5], right trapezius 1.7 [SD 2.4]). Sensitivity was significantly greater at the target joint than at the left (*P* = 0.007) and right trapezius (*P* = 0.000). L, left; MDT, mechanical detection threshold; MPT, mechanical pain threshold; PPT, pressure pain threshold; QST, quantitative sensory testing; R, right.

Cutaneous sensation (MDT) and pain thresholds (MPT) showed no deviation from normative values at the target joint or control site (Figure 2A, Z values < 1.96). There was a significant increase in sensitivity in MPT at the control site compared to the target joint (*P* = 0.006), which may reflect a normal difference in sensation between the joint and forearm sites or hypoesthesia due to inflammation at the joint (Supplementary Table S2). Comparison with healthy controls would be required to determine this.

#### Static QST: PPT at target joint and trapezius

A low PPT reflects pain sensitization, and a higher Z score is indicative of gain of function (sensitivity), with a Z score of ≥1.96 representing significant sensitivity. As a group, participants showed significantly increased sensitivity compared to normative data at all sites except the right trapezius (mean PPT Z score at target joint 3.8 [SD 3.0], left wrist 2.9 [SD 3.1], right wrist 2.9 [SD 2.5], left trapezius 2.1 [SD 2.5], right trapezius 1.7 [SD 2.4]; Supplementary Table S3). Sensitivity was significantly greater at the target joint than at the left (*P* = 0.007) and right trapezius (*P* = 0.000). Sixteen (26%) had significant sensitivity (Z score ≥ 1.96) at joint sites only, suggesting peripheral sensitization. Thirty‐four (56%) had significant sensitivity at both joint and trapezius sites, indicating possible widespread centrally mediated pain. No patients had trapezius sensitivity without joint or wrist sensitivity. Eleven (18%) showed no significant sensitivity at any site.

#### Dynamic QST: TSP

**Figure 3:**
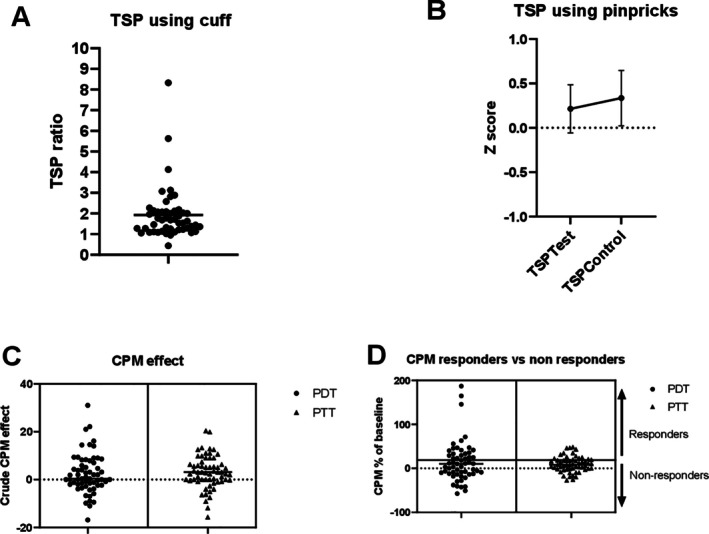
Graphs showing results from dynamic QST. (A) Individual values for the TSP using cuff (n = 51). Nine of fifty‐one (18%) fulfilled the criteria for facilitated TSP, with a ratio of >2.48. (B) Z score for the study population TSP using pinpricks (n = 51). There was no significant difference in TSP using pinpricks compared to normative data, reflected by Z scores <1.96. (C) Graph showing individual values of crude CPM effect (PDT/PTT_(CPM)_ − PDT/PTT_(Dom)_) for PDT and PTT (n = 57). (D) Graph showing individual values of CPM effect as a percentage of baseline for PDT and PTT. Participants were stratified as responders (CPM response >20% of the baseline cPPT) or nonresponders (CPM response ≤20% of baseline cPPT) (n = 57). According to PDT and PTT, 35 of 57 (61%) and 44 of 57 (77%), respectively, were nonresponders. CPM, conditioned pain modulation; cPPT, cuff pressure pain threshold; Dom, Dominant leg; PDT, pain detection threshold; PTT, pain tolerance threshold; QST, quantitative sensory testing; TSP, temporal summation of pain.

Using the cuff algometer, 9 of 51 (18%) fulfilled criteria for facilitated TSP with a ratio of >2.48 (Figure 3A and Supplementary Figure S1). In contrast, there was no difference in TSP, using pinpricks compared to normative data at either the target joint (test) or the control site (Figure 3B).

#### Dynamic QST: CPM

In total, 35 of 57 participants (61%) were CPM nonresponders (ie, those not reporting a decrease in pain perception upon the application of a painful test stimulus concurrent to a conditioning stimulus) based on pain detection thresholds, and 44 of 57 (77%) were CPM nonresponders based on pain tolerance thresholds (Figure 3C and D). Although pain detection thresholds did not vary across CPM nonresponder and responder groups, CPM nonresponders reported lower pain tolerance thresholds (Supplementary Table S4), highlighting that although both groups had similar initial pain sensitivity, nonresponders had a reduced ability to endure pain.

### Multivariable linear regression model

In blockwise linear regression, MSKUS total GS and PD alone (block 1), our measure of peripheral inflammation, explained little of the variance in pain scores (R^2^ = 0.0006, *P* = 0.86). Adding age and sex (block 2) modestly increased the explained variance (R^2^ = 0.0619, *P* = 0.36). In the final model (block 3), which included additional variables plausibly linked to pain, fatigue, mood (PHQ Anxiety–Depression Scale), fibromyalgia severity (WPI and SSS), painDETECT score, and trapezius PPT, the explained variance increased substantially (R^2^ = 0.3538, *P* = 0.009). Contrary to our hypothesis that inflammation would be a key driver of pain, MSKUS total GS and PD contributed little to the variance in pain scores, suggesting that noninflammatory factors may contribute more to pain severity in this cohort.

### Correlations and factor analysis of clinical outcome measures and PROMs

Clinical and patient‐reported outcomes that suggest centrally mediated pain and lower nonarticular PPT correlated with higher disease activity (DAS28), increased fatigue, decreased QoL, greater impact of disease, and worse mental health measures (Table 3). In contrast, clinical measures of peripheral inflammation (total GS and PD positivity on ultrasonography, SJC) did not (Table 3). There was no correlation between clinical markers of central pain and dynamic QST (Supplementary Table S5).

**Table 3:** Correlation between markers of centrally mediated pain/peripheral inflammation and clinical parameters*

	DAS28	TJC‐28	SJC‐28	Time since diagnosis	Symptom duration	Fatigue	MSK‐HQ	RAID	GAD‐7	PHQ‐9	PHQ‐15
Markers of centrally mediated pain											
Total pain	0.513^a^ ^,^ ^e^	0.394^b^ ^,^ ^e^	0.006^d^	−0.103^c^	0.116^c^	0.460^b^ ^,^ ^e^	−0.475^b^ ^,^ ^e^	0.58^a^ ^,^ ^e^	0.301^b^ ^,^ ^e^	0.307^b^ ^,^ ^e^	0.386^b^ ^,^ ^e^
Fibromyalgia severity (WPI + SSS)	0.532^a^ ^,^ ^e^	0.524^a^ ^,^ ^e^	−0.093^d^	−0.153^c^	0.039^d^	0.647^a^ ^,^ ^e^	−0.653^a^ ^,^ ^e^	0.684^a^ ^,^ ^e^	0.636^a^ ^,^ ^e^	0.685^a^ ^,^ ^e^	0.674^a^ ^,^ ^e^
painDETECT	0.514^a^ ^,^ ^e^	0.454^b^ ^,^ ^e^	−0.132^c^	−0.175^c^	−0.073^d^	0.501^a^ ^,^ ^e^	−0.5^a^ ^,^ ^e^	0.596^a^ ^,^ ^e^	0.501^a^ ^,^ ^e^	0.493^b^ ^,^ ^e^	0.681^a^ ^,^ ^e^
PPT at the trapezius (average)	−0.293^c^	−0.267^c^	0.082^d^	0.043^d^	0.098^d^	−0.301^b^	0.333^b^ ^,^ ^e^	−0.432^b^	−0.188^c^	−0.295^c^	−0.286^c^
Markers of peripheral inflammation											
SJC	0.337^b^ ^,^ ^e^	0.207^c^	–	0.038^d^	0.077^d^	0.022^d^	−0.112^c^	0.032^d^	−0.243^c^	−0.191^c^	−0.125^c^
Total GS and PD positivity	−0.106^c^	−0.007^d^	0.546^a^ ^,^ ^e^	−0.019^d^	−0.001^d^	−0.021^d^	−0.036^d^	−0.039^d^	−0.210^c^	−0.222^c^	−0.199^c^

* Correlation analysis for measures of centrally mediated pain and peripheral inflammation with clinical parameters. Each cell displays the correlation coefficient between a pain or inflammation marker (rows) and a clinical variable (columns). Numerical correlation coefficients are annotated within each cell. Higher total pain, fibromyalgia severity, and painDETECT scores and lowered PPT at the bilateral trapezius (taken as an average of both sides) correlated with higher disease activity (DAS28) and TJC but not SJC. They also correlated with increased fatigue, decreased QoL (MSK‐HQ), and greater impact of disease (RAID). Additionally, these measures showed correlations with mental health measures, including higher levels of anxiety (GAD‐7), depression (PHQ‐9), and somatic symptoms (PHQ‐15) (with the exception of PPT at the trapezius and anxiety). Notably, these measures did not demonstrate correlations with either time since diagnosis or symptom duration. Total GS and PD positivity in the 14‐joint ultrasonography assessment represents the combined scores of GS and PD assessments. The SJC and total GS and PD positivity serve as indicators of peripheral inflammation, which contributes to peripheral pain. SJC correlated with disease activity (DAS28), and total GS and PD positivity correlated with SJC. However, neither the SJC nor total GS and PD positivity correlated with TJC, fibromyalgia severity, QoL by MSK‐HQ or RAID, or mental health measures (GAD‐7, PHQ‐9, or PHQ‐15). DAS28, Disease Activity Score in 28 joints; GAD‐7, Generalized Anxiety Disorder 7; GS, gray scale; MSK‐HQ, Musculoskeletal Health Questionnaire; PD, power Doppler; PHQ, Patient Health Questionnaire; PPT, pressure pain threshold; QoL, quality of life; RAID, Rheumatoid Arthritis Impact of Disease; SJC, swollen joint count; SSS, Symptom Severity Scale; TJC, tender joint count; WPI, Widespread Pain Index.

^a^ Strong correlations (|r| ≥ 0.5).

^b^ Moderate correlations (|r| = 0.3–0.49).

^c^ Weak correlations (|r| = 0.1–0.29).

^d^ Negligible correlations (|r| < 0.1).

^e^ Statistical significance at the *P* < 0.05 level.

Based on factor analyses of the 14 variables, we identified two underlying latent factors. CRP, TSP, and CPM had Kaiser–Meyer–Olkin (KMO) statistics below 0.5 and were not included in the model. The overall KMO statistic was 0.76, indicating the correlation matrix of the remaining variables was appropriate for factor analysis.^35^ Factor 1 included self‐reported variables of pain (total pain, patient global assessment, TJC, painDETECT, fibromyalgia criteria, and trapezius PPT) and fatigue and mental health (PHQ‐9 and GAD‐7). Factor 2 included clinical and ultrasonographic measures of synovitis (SJC, GS and PD on MSKUS, and physician global assessment; Supplementary Table S6). The correlation between the two factors was −0.13.

## DISCUSSION

This study provides novel insights into the mechanisms underlying pain reported by patients with early IA, as we compiled comprehensive sensory profiles of individuals shortly after diagnosis. Consistent with newly diagnosed IA, 97% of patients had active synovitis, confirming that peripheral inflammation is highly prevalent at this stage and likely contributes to pain. However, we also observed indicators of centrally mediated pain in approximately 20% to 30% of patients who reported widespread pain, fulfilled diagnostic criteria for fibromyalgia, and/or demonstrated elevated painDETECT scores. QST findings further supported the involvement of centrally mediated pain mechanisms. Reduced PPTs at nonarticular sites in some patients suggested widespread hyperalgesia, whereas heightened responses to TSP and impaired CPM indicated dysfunction in spinal and supraspinal processes. The regression analysis showed that inflammation alone explained very little variance in total pain scores, and it was centrally mediated pain factors that correlated more strongly with clinical outcomes such as QoL and impact of disease. This suggests that although inflammation may trigger nociceptive input, other factors, including central mechanisms driven by, for example, psychosocial factors, play a substantial role in shaping individual pain experiences even early in the disease course. Overall, these data challenge the longstanding assumption that only peripheral mechanisms driven by joint inflammation underpin pain at diagnosis. Instead, they suggest that some patients with early IA have both inflammatory and centrally mediated pain, similar to patients with established IA.^5^, ^6^, ^14^

Our cohort's clinical disease activity measures and average pain score of 5.5 of 10 aligns with findings reported in other early IA cohorts,^36^ as do the comparably high rates of anxiety and/or depression.^37^ Although pain mechanisms in early IA remain relatively unexplored, our findings revealed that 21% of patients met the criteria for fibromyalgia. Some of these cases may represent individuals with underlying primary fibromyalgia. However, this figure is much higher than the estimated prevalence of 1.78% in the general population.^38^ In addition, the mean symptom duration of six months would allow sufficient time for, for example, centrally mediated pain to develop in response to peripheral nociceptive input. It also exceeds the 5.2% prevalence reported in one Canadian study.^39^ This may reflect methodologic differences, with the Canadian study relying on clinical judgment rather than strict fibromyalgia diagnostic criteria or the Canadian cohort having a more inflammatory profile than ours or other early IA cohorts.^40^, ^41^ In addition, symptoms of very active early IA, such as widespread pain, fatigue, and unrefreshing sleep, overlaps with fibromyalgia criteria, potentially resulting in a “false” fibromyalgia diagnosis. However, the lack of correlation between WPI/fibromyalgia severity and peripheral inflammation markers suggests that the observed widespread pain may be driven by mechanisms other than joint inflammation.

We observed reduced PPT values at the affected joint in most patients, indicating local pain sensitization, likely due to joint inflammation, in this early arthritis cohort. In addition, we found lowered PPT values at nonarticular sites, indicative of widespread pain, in some patients. These findings are in keeping with those from a recent meta‐analysis of patients with established IA^14^ but are the first to identify such reductions in PPT at nonarticular sites specifically in the context of early IA, highlighting the presence of widespread pain sensitivity in some patients early in the disease course. Factor analysis revealed close links between reduced PPT at nonarticular sites and self‐reported pain, fatigue, and poor mental health, which are often associated with centrally mediated pain. The lack of interaction with dynamic QST (TSP or CPM) measures suggests that reduced PPT at nonarticular sites may be the most robust QST indication of centrally mediated pain in this patient population. Although the analysis was used primarily as a data reduction technique rather than to identify a replicable latent structure, it was conducted on a relatively small sample. This limits the stability and generalizability of the resulting factor structure.

Eighteen percent of participants demonstrated facilitated TSP, in which a TSP paradigm with the cuff algometer was used as a repetitive stimulus, indicating spinal mechanisms contributing to heightened pain. However, when pinpricks were used as the repetitive stimulus, facilitated TSP was not observed, contrasting with findings from a study that also used pinpricks in patients with established IA.^42^ This difference in findings could be attributed to characteristics specific to our study population—ours was a recently diagnosed patient cohort with IA—or, alternatively, due to methodologic protocols. For example, the initial pinprick force may not have been sufficiently noxious, or the initial pinprick rating may have been too high, creating a ceiling effect on subsequent pain ratings. When applying a CPM paradigm, 61% and 77% of the participants exhibited a “nonresponder status,” according to pain detection threshold and pain tolerance threshold, respectively, indicating dysfunction in a naturally occurring pain inhibitory pathway. Although data are lacking to compare the proportion of nonresponders with healthy controls,^29^ our cohort's mean CPM effect for pain detection threshold was 2.4, below that found in healthy populations, in which the mean CPM effect for pain detection threshold ranges^30^ from 5.3 to 11.5.

This study has several strengths. The in‐depth sensory profiling allowed us to create a comprehensive profile, combining measures of pain sensitivity and centrally mediated pain‐processing mechanisms and ultrasonography as an objective measure of joint inflammation. All assessments were performed by a single researcher, ensuring consistency and minimizing interrater variability. We attempted early assessment of patients soon after their diagnosis, which was usually achieved, enabling insights into what is driving pain in the very early stages of IA soon after diagnosis.

The study also has limitations. One key limitation is that there is currently no gold standard comprehensive method for assessing centrally mediated pain. As a result, instruments such as questionnaires and QST, which have been validated individually, are combined. However, these instruments are frequently not standardized or validated in comparison to each other. For example, although used in the literature as a proxy measure of centrally mediated pain, there is currently limited data linking “likely neuropathic pain” identified by painDETECT to QST findings in IA.^43^ Furthermore, as PPT normative data for the wrist and trapezius were not available, data from the thenar eminence (a potentially more sensitive site) were used for comparison. Because PPT varies by anatomic site, this may have led to systematically inflated Z scores at the wrist and trapezius and could partly explain the high proportion of participants showing sensitivity. Alternatively, it is possible that PPT is a more sensitive measure of pain processing than PROMs.

Another important consideration is that the enrollment criteria specified pain levels of ≥3. This threshold was chosen to align with the study's aim of exploring pain mechanisms in early IA. However, these criteria may limit the generalizability of results to those people in at least moderate pain, and it is likely that, in the absence of this threshold, the proportion of participants meeting criteria for centrally mediated pain would be lower. Additionally, given the relatively short time since diagnosis, it is possible that some participants would not go on to develop chronic IA, which would limit the generalizability of the results. At the time of study assessment, most patients had already received therapy, and although we captured patients within a short time post diagnosis, most individuals had pain in the period before diagnosis, and so in some individuals, central nervous system dysfunction may have predated joint inflammation. It has been postulated that a preloading psychological vulnerability to pain may contribute to the development of persistent noninflammatory pain. An alternative explanation is that this could also be influenced by subclinical joint pathology. We will follow this group with longitudinal investigations aimed at identifying evolution of pain mechanisms over time and possible signals for those who might develop persistent noninflammatory pain.

These findings emphasize that pain in early IA is complex and, in some people, includes centrally mediated pain mechanisms, highlighting the need for awareness of these factors in early IA populations. Future longitudinal studies are essential to track the evolution of pain mechanisms over time and identify predictors of persistent pain, which may inform early interventions targeting central pain processes.

## AUTHOR CONTRIBUTIONS

All authors contributed to at least one of the following manuscript preparation roles: conceptualization AND/OR methodology, software, investigation, formal analysis, data curation, visualization, and validation AND drafting or reviewing/editing the final draft. As corresponding author, Dr Rutter‐Locher confirms that all authors have provided the final approval of the version to be published and takes responsibility for the affirmations regarding article submission (eg, not under consideration by another journal), the integrity of the data presented, and the statements regarding compliance with institutional review board/Declaration of Helsinki requirements.

## Supporting information


**Disclosure Form**:

**Data S1** Supporting Information
