# Supplementary material for: Assessment of Pain Types in Recently Diagnosed Patients With Inflammatory Arthritis
Source: Arthritis Care Res (Hoboken). 2025 Oct 7;78(1):154–63. doi: 10.1002/acr.25651 (PMC12826086; doi:10.1002/acr.25651)
Supplement: Supplementary file 2 — Data S1 Supporting Information [file ACR-78-154-s002.docx]

Supplementary material:

Methods

Dynamic QST protocols

Temporal summation of pain (TSP) using cuff pressure algometer

The test leg cuff was inflated for 1 second, repeated 10 times at 1-second intervals, at a pressure equivalent to 100% of the participant's pressure pain tolerance in that leg. Participants were asked to rate the pain experienced during each compression using a visual analog scale (VAS). TSP was determined by calculating the ratio of VAS III (mean VAS for stimulations 8-10) to VAS I (mean VAS for stimulations 1-4), where TSP is considered to be facilitated if the ratio exceeds 2.48 (28).

Conditioned pain modulation (CPM) using cuff pressure algometer

Two blood pressure cuff-like devices placed around the widest part of the gastrocnemius muscle on the lower leg act as the test stimulus (dominant leg) and conditioning stimulus (non-dominant leg). These were inflated at a computer-controlled steady rate of 1kPa/second using compressed air. The subject was given a visual analogue scale bar and asked to begin to slide the bar at first sensation of pain. The pressure value when the patient rates 1cm on the VAS is the pain detection threshold (PDT) and when the patient can no longer tolerate the pressure they press the stop button to immediately deflate the cuff (pain tolerance threshold, PTT). Firstly, the PDT and PTT were assessed on the test leg, and then CPM was assessed as the PDT and PTT in the test leg with the conditioning leg concurrently inflated (at 70% of the PTT). CPM response was defined as PPT CPM effect = PPT_(CPM)_- PPT _(Dom)_ and PTT CPM effect = PTT_(CPM)_- PTT _(Dom)._ Participants were stratified as responders (CPM effect > 20% of the baseline cPPT) or non-responders (CPM effect ≤20% of baseline cPPT) (29)[12][27]

Factor analysis

A set of 14 variables was selected for potential inclusion. Where variables were not approximately normally distributed, they were transformed by taking the natural logarithm or square root. Items with Kaiser-Meyer-Olkin (KMO) measures of sampling adequacy < 0.5 were excluded. An overall KMO level >0.6 was considered to indicate the set of variables was appropriate for factorisation. The number of factors was determined based on parallel analysis and visual inspection of a scree plot. Oblimin rotation was employed to facilitate the interpretation of the factor structure and allow the factors to correlate. Factor loadings above 0.40 were considered to indicate that the variable 'loaded' onto a factor. That is, the factor explained a meaningful amount of the variance in that variable.

Results

Clinical and patient-reported outcome measures (PROMs)

| **Variable** | **PAINDETECT** | **WPI Score** | **T–SJC** | **FM Severity** |
| --- | --- | --- | --- | --- |
| **PAINDETECT** | — |  |  |  |
| **WPI Score** | .36 (p = .004) | — |  |  |
| **T–SJC** | .50 (p < .001) | .42 (p < .001) | — |  |
| **FM Severity** | .59 (p < .001) | .81 (p < .001) | .54 (p < .001) | — |

# Supplementary Table S1 : Correlation analysis between measures of central pain: painDETECT, WPI score, T-SJC and fibromyalgia severity (sum of WPI and SSS). Correlation coefficients are in the moderate to strong range, with all associations statistically significant (p < 0.05).

# Static and dynamic QST findings

Here we present the QST findings in more detail.

**Cutaneous sensation and pain thresholds were normal**

Skin sensitivity was assessed using mechanical detection threshold (MDT) and mechanical pain threshold (MPT) according the DFNS protocol. At the test site, the mean z-score for MDT was 0.07 ± 1.9, and at the control site, it was 0.03 ± 1.6. For MPT, the mean z-score at the test site was 0.26 ± 1.7, and at the control site, it was 1.09 ± 1.7. This indicates as a population there was no difference between the participants and the age- and gender-matched healthy control data, either at the target joint or the contralateral forearm (Z scores below 1.96, indicating no significant deviation) (Figure 2A in main manuscript).

The proportion of those with gain in function (hypersensitivity) measured by MPT at the control site was higher than at the test site (Supplementary Table S2). Otherwise, approximately 15% of patients experienced increase in function (hypersensitivity) or decrease in function (hypoalgesia) in MDT and MPT at the test and control sites.

Supplementary Table S2: The proportion of those with significant gain in function (Z score >1.96) or decrease in function (Z score <-1.96) for MDT and MPT at each site.

|  | Gain in function (n, %) | Decrease in function (n, %) |
| --- | --- | --- |
| MDT Test | 12 (20%) | 10 (16%) |
| MDT Control | 8 (13%) | 9 (15%) |
| MPT Test | 10 (16%) | 9 (15%) |
| MPT Control | 21 (34%) | 5 (8%) |

**Target painful joints have low PPT, but also some patients have low PPT at the trapezius**

Pressure pain thresholds (PPT) were measured at the target joint, with low PPT reflective of peripheral sensitisation. PPT data was then taken at sites distant from the joint, specifically the bilateral trapezius muscle, with lowered PPT reflective of proposed widespread hyperalgesia caused by central mechanisms.

As a group, participants showed significantly increased sensitivity at all sites except the right trapezius (mean PPT Z score at: target joint: 3.8 [SD 3.0], left wrist: 2.9 [3.1], right wrist: 2.9 [2.5], left trapezius: 2.1 [2.5], right trapezius: 1.7 [2.4]). Sensitivity was significantly greater at the target joint than at the left (p=0.007) and right trapezius (p=0.000). 16 (26%) were significantly sensitive at joint sites only, suggesting peripheral sensitisation. 34 (56%) had sensitivity at both joint and trapezius sites, indicating possible widespread centrally mediated pain. No patients had trapezius sensitivity without joint/wrist sensitivity. 11 (18%) showed no sensitivity at any site.

Supplementary Table S3: PPT (kg/cm^2^) at joint sites and sites distant from the joint, in this case the trapezius. (n=60)

| **Site** | **Mean ±SD PPT(kg)** |
| --- | --- |
| Target joint (n=60) | 2.2 ±1.6 |
| L wrist (n=57) | 2.7 ±1.7 |
| R wrist (n=55) | 2.5 ±1.2 |
| L trapezius (n=54) | 3.1±1.8 |
| R trapezius (n=60) | 3.3 ±2.0 |

**Temporal summation of pain was evident using cuff algometer but not pinprick**

Using pinpricks, the mean WUR was 2.9±2.1 at the test site and 3.3±2.8 at the control site. The mean z-score for WUR at the test site was 0.22 ±0.98 and control site was 0.33 ±1.1. Therefore, there was no significant difference between mean WUR at either test or control site compared to age and gender matched healthy reference data (Figure 3A in main manuscript). 3 (5%) of participants had abnormal TSP at the control site and 1 (2%) at the test site (Z score >1.96).

Using the cuff algometer, meanTSP ratio was 1.93 ±1.3, excluding an outlier of 59.6 (Mean VAS 8-10: 1.49, mean VAS 1-4: 0.025), (Supplementary Figure S1). 9/51 (18%) fulfilled criteria for facilitated TSP, with a ratio of >2.48 (Figure 3B in main manuscript).

Supplementary Figure S1: Line graph showing mean VAS rating for subsequent cuff inflation. The mean TSP ratio was 1.93 ±1.3, excluding an outlier of 59.6 (Mean VAS 8-10: 1.49, mean VAS 1-4: 0.025).

**Abnormal response to CPM was evident in most participants**

Mean PDT for the test leg was 20.9±11. Mean PDT for the test leg with conditioning rose to 23.3 ±15. Therefore, the mean CPM effect for PDT was 2.4±8.3.

Mean PTT for the test leg was 38.9±18, with mean VAS 6 ±2.9. Mean PTT for the test leg with conditioning rose to 42.0±19, with mean VAS 5.7±2.9. Therefore, the mean CPM effect for PTT was 3.1 ±6.8.

35/57 (61%) according to PDT and 44/57 (77%) according to PTT were non-responders and had impaired CPM (Figure 3C&D main manuscript). Although there was no difference in test leg PDT in responders as compared to non-responders, non-responders had significantly lower test leg PTT. Non-responders also had significantly lower PDT and PTT in the test leg with conditioning than responders (Supplementary Table S4).

Participants with painDETECT≥19 had lower test leg PTT, but not PDT, than those with painDETECT<19 (Mean PDT with high painDETECT 16±12 and with low painDETECT 22±11,p=0.062, Mean PTT with high painDETECT 28±19 and with low painDETECT 42±17,p=0.007). Participants fulfilling fibromyalgia criteria also had lower test leg PTT, but not PDT, than those without fibromyalgia (Mean PDT with fibromyalgia 17±13 and without fibromyalgia 22±11, p=0.198, Mean PTT with fibromalgia 29±19 and without fibromyalgia 42±17, p=0.023)

Supplementary Table S4: PDT and PTT values in responders versus non responders to the CPM paradigm

|  | Non-Responders | Responders | Significance |
| --- | --- | --- | --- |
| Test leg PDT | 19.8±11.4 | 22.8±11 | P=0.34 |
| Test leg PTT | 35.0±18 | 45.6±17 | P=0.03* |
| Test leg PDT with conditioning | 17.6±11 | 33.5±15 | P=0.00* |
| Test leg PTT with conditioning | 35.5±18 | 53.5±17 | P=0.00* |

**There was no correlation between TS/CPM and disease activity, QoL or mental health**

There was no correlation between TSP (WUR at test site/ Cuff ratio) or CPM for PDT/PTT with clinical markers including: DAS28, TJC, SJC, time since diagnosis, symptom duration, fatigue, MSK-HQ, RAID, GAD 7, PHQ9 or PHQ15 (excluding Cuff ratio and TJC, 0.45 (p=0.001) (Supplementary Table S5).

There was also no significant difference in means of clinical markers (pain, T/SJC, DAS28, MSKHQ, RAID, Mental health, fatigue, painDETECT, Fibromyalgia severity) between those with facilitated vs normal TSP and responders vs non responders to CPM.

**There was a correlation between the clinical markers of central pain, but not between these clinical markers and QST measures**

There was a correlation between the clinical markers of central pain (total pain, fibromyalgia severity and pain DETECT, fatigue). There was also a correlation between total pain, fibromyalgia criteria and fatigue with PPT at the trapezius, but this was not significant for painDETECT. Broadly, there was no correlation between clinical markers of central pain (total pain, fibromyalgia severity, painDETECT, fatigue) and dynamic QST (TSP or CPM) (Supplementary Table S5).

Supplementary Table S5: Correlation between QST parameters and clinical parameters. *p<0.05

|  | **Total pain** | **Fibromyalgia severity (WPI + SSS)** | **Fatigue** | **painDETECT** | **PPT at trapezius** | **WUR (pinprick)** | **TSP ratio (Cuff)** | **CPM (PDT)** | **CPM (PTT)** |
| --- | --- | --- | --- | --- | --- | --- | --- | --- | --- |
| **Total pain** | **1** | **0.338*** | 0.460* | 0.354* | -0.295* | 0.114 | **-0.030** | **-0.365*** | **-0.236** |
| **Fibromyalgia severity (WPI+SSS)** | **0.338*** | **1** | 0.647* | 0.592* | -0.297* | -0.041 | **0.042** | **-0.1.06** | **-0.042** |
| **Fatigue** | **0.460*** | **0.647*** | 1 | 0.501* | -0.301* | 0.022 | **-0.019** | **-0.151** | **0.007** |
| **PainDETECT** | **0.354*** | **0.592*** | 0.501* | 1 | -0.233 | 0.038 | **0.136** | **-0.228** | **-0.152** |
| **PPT at the trapezius (average)** | **-0.295*** | **-0.297*** | -0.301* | -0.233 | 1 | 0.207 | **-0.041** | **0.487*** | **0.261** |
| **WUR (pinprick)** | **0.114** | **-0.041** | 0.022 | 0.038 | 0.207 | 1 | **0.052** | **0.114** | **-0.051** |
| **TSP ratio (Cuff)** | **-0.03** | **0.042** | -0.019 | 0.136 | -0.041 | 0.052 | **1** | **-0.016** | **0.023** |
| **CPM (PDT)** | **-0.365*** | **-0.106** | -0.151 | -0.228 | 0.487* | 0.114 | **-0.016** | **1** | **0.495*** |
| **CPM (PTT)** | **-0.236** | **-0.042** | 0.007 | -0.152 | 0.261 | -0.051 | **0.023** | **0.495*** | **1** |

# Factor analysis

Supplementary Table S6: Results of factor analysis showing two factors: Factor 1 included self-reported variables on pain (total pain, patient global assessment, TJC, painDETECT, fibromyalgia criteria and trapezius PPT), fatigue and mental health (PHQ 9 and GAD 7). Factor 2 included clinical assessments of synovitis (SJC, GS and PD on MSK US and physician global assessment) Factor loadings above 0.40 were considered significant.

| **Variable** | **Factor 1** | **Factor 2** | **Uniqueness** |
| --- | --- | --- | --- |
| Fatigue | 0.79 |  | 0.36 |
| Total pain | 0.53 |  | 0.60 |
| painDETECT | 0.68 |  | 0.53 |
| TJC | 0.50 | 0.46 | 0.49 |
| SJC |  | 0.78 | 0.39 |
| MSK US |  | 0.42 | 0.80 |
| MSK HQ | -0.80 |  | 0.29 |
| Patient global | 0.76 |  | 0.23 |
| Physician global |  | 0.70 | 0.45 |
| Fibromyalgia severity | 0.84 |  | 0.30 |
| PPT at trapezius | -0.43 |  | 0.81 |
| PHQ 9 (depression) | 0.82 |  | 0.30 |
| GAD 7 (anxiety) | 0.77 |  | 0.37 |
